# “Slipped capital femoral epiphysis in a 25-year-old hypogonadic man with a large cranial chondroma: causality or coincidence? “

**DOI:** 10.1186/s12902-021-00828-0

**Published:** 2021-08-17

**Authors:** Nadia Sawicka-Gutaj, Waldemar Woźniak, Jakub Naczk, Mateusz Pochylski, Jacek Kruczyński, Bartłomiej Budny, Ewelina Szczepanek-Parulska, Marek Ruchała

**Affiliations:** 1 Department of Endocrinology, Metabolism and Internal Medicine, Poznan University of Medical Sciences, Poznan, Poland; 2 Department of General and Oncology Orthopaedics and Traumatology, Poznan University of Medical Sciences, Poznan, Poland

**Keywords:** Hip, Slipped capital femoral epiphysis, Tumor, Chondroma, Hypogonadism

## Abstract

**Background:**

Slipped capital femoral epiphysis (SCFE) is a hip disorder frequently occurring in adolescence. In adults it is rare and so far very few cases have been documented.

**Case presentation:**

This report presents a 25-year-old patient diagnosed with an anterior fossa giant chondroma, hypogonadotropic hypogonadism, and SCFE. The patient underwent surgical and hormonal therapy. His symptoms revealed, and he became a father.

**Conclusions:**

Every patient diagnosed with SCFE in adulthood should undergo endocrinological assessment based on physical examination and laboratory tests.

## Background

Slipped capital femoral epiphysis (SCFE) is a frequent hip disorder in adolescence, which should be quickly diagnosed and treated. The sub-acute and long-term consequences of SCFE are the loss of joint function and osteoarthritis due to femoroacetabular impingement [1].

SCFE affects about 10.8 in 100,000 people [2]. Despite many theories, its aetiology is still unclear. Incorrect distribution of forces working on the proximal part of the femur, relative and absolute retroversion of the femoral neck are identified as potential mechanical factors which cause SCFE. Obesity [3–5], endocrine disorders (hypothyroidism, hypopituitarism, hypoparathyroidism) [5, 6], genetic disorders [7, 8] and growth hormone therapy are potential causes of epiphyseal cartilage injury. Slipped capital femoral epihysis in adults is very rare. So far very few cases have been documented. This article presents a 25-year-old patient diagnosed with an anterior fossa giant chondroma, hypogonadotropic hypogonadism, and SCFE.

## Case presentation

**Fig. 1:**
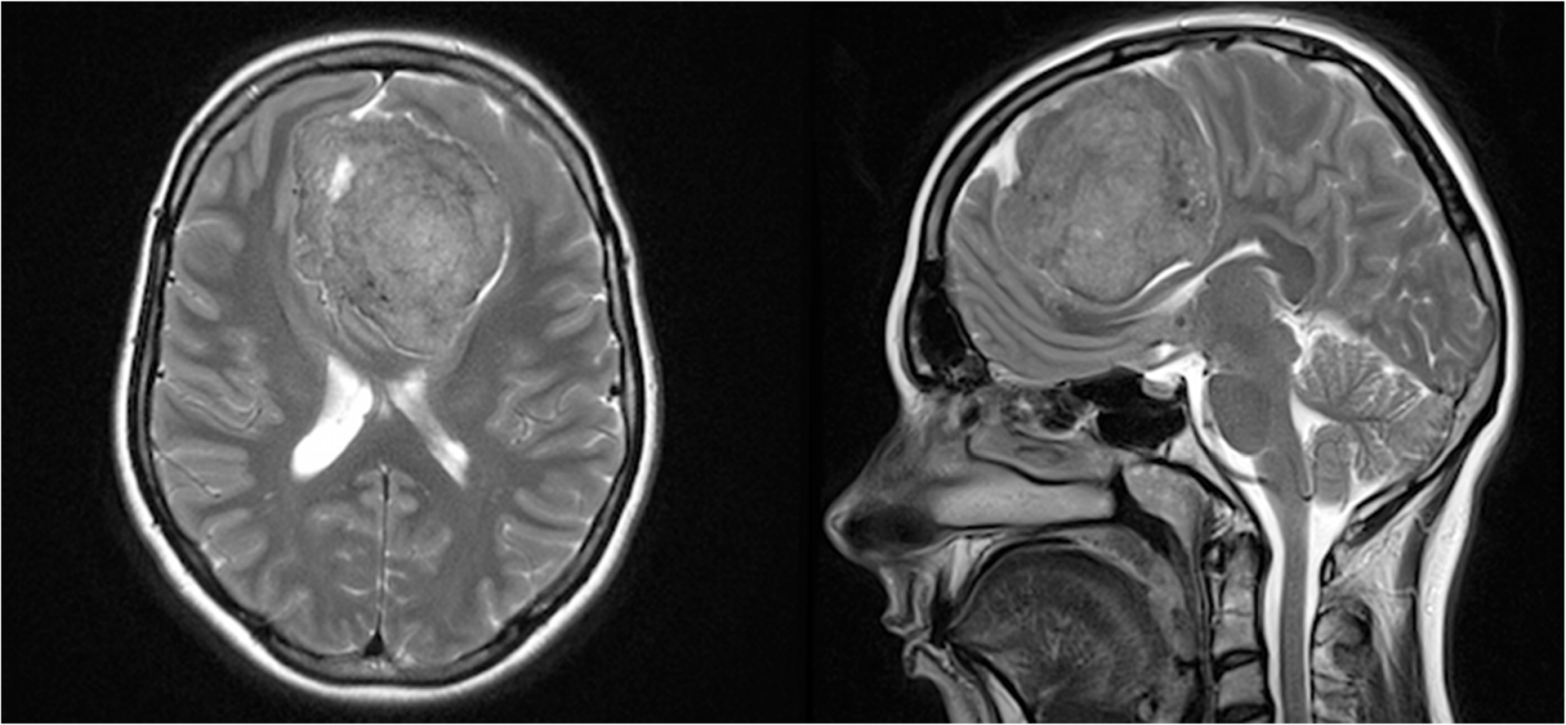
Anterior cranial fossa tumor

So far apparently healthy 25-year-old white male presented to an emergency room with a chief complaint of tonic seizures. The diagnostic imaging (NMR of the head and angio-CT) showed that the man suffered from a large anterior cranial fossa tumour (Fig. 1).

The tumor was removed by the fronto-parietal craniotomy. Histopathological examination revealed it was a chondroma. The postoperative period was uneventful.

**Fig. 2:**
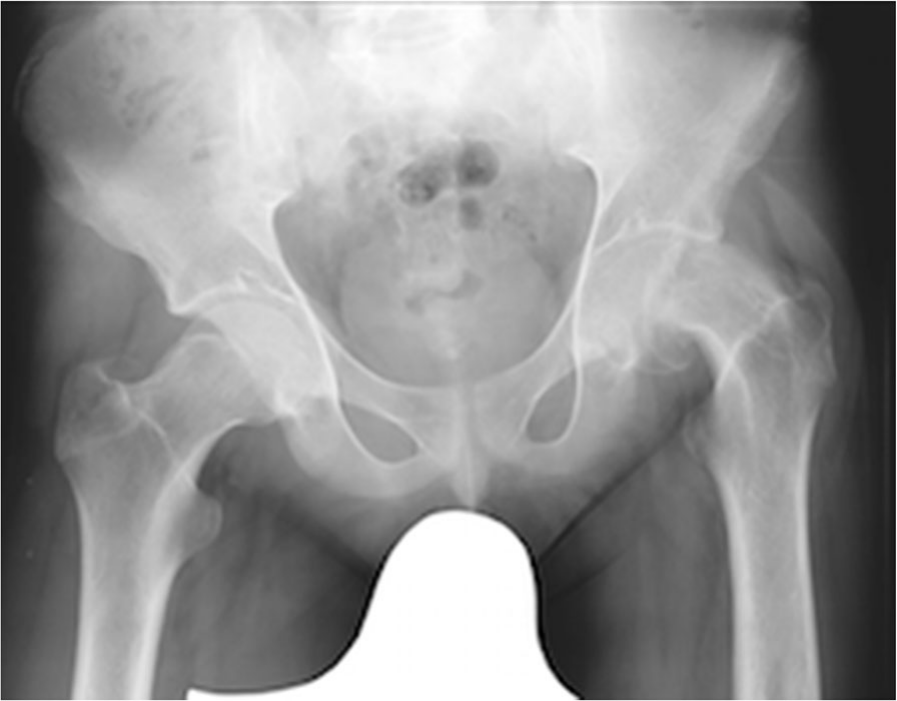
XR A/P of the hip

**Fig. 3:**
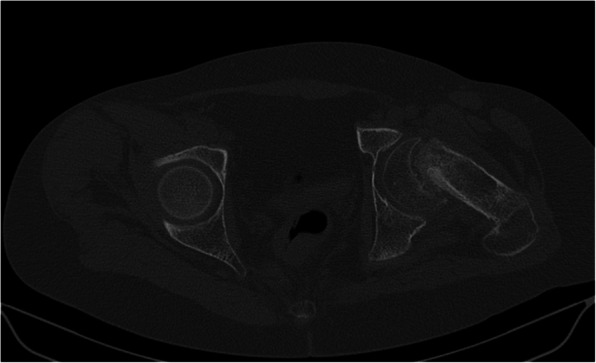
SCFE in transverse plane CT before surgery

**Fig. 4:**
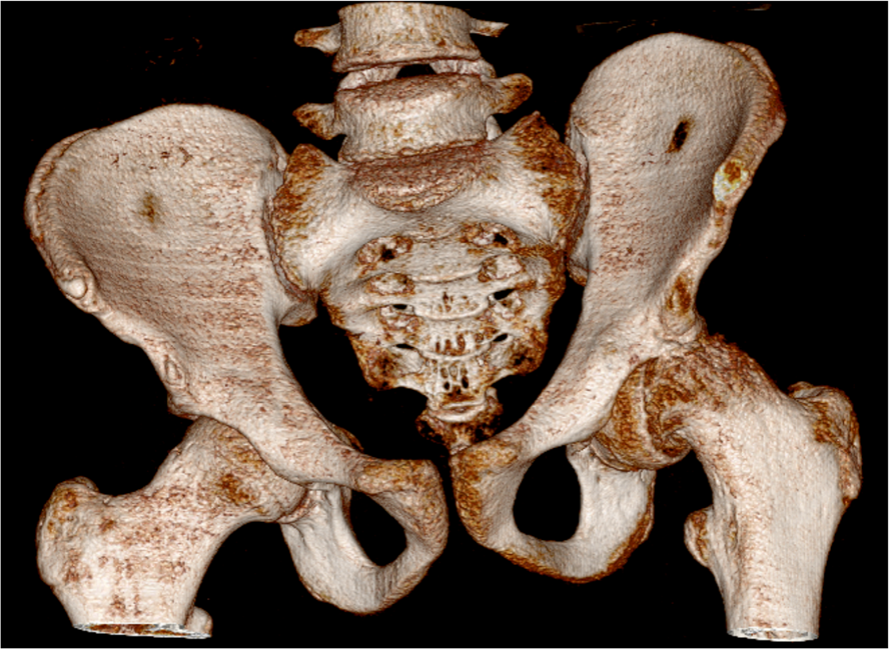
Hip CT 3D-reconstruction before surgery

**Fig. 5:**
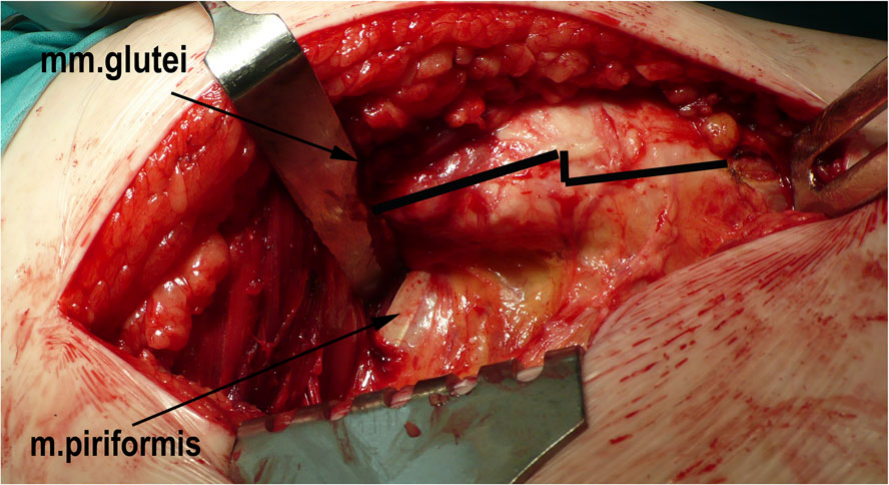
Osteotomy of the greater trochanter

**Fig. 6:**
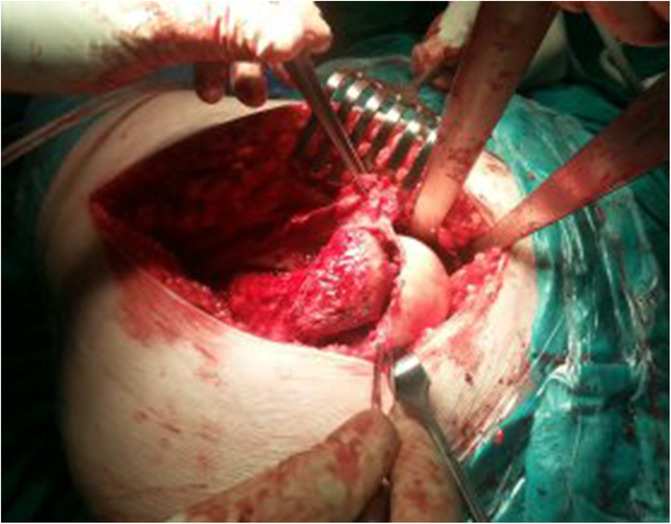
Separation of the head of the femur from the neck

**Fig. 7:**
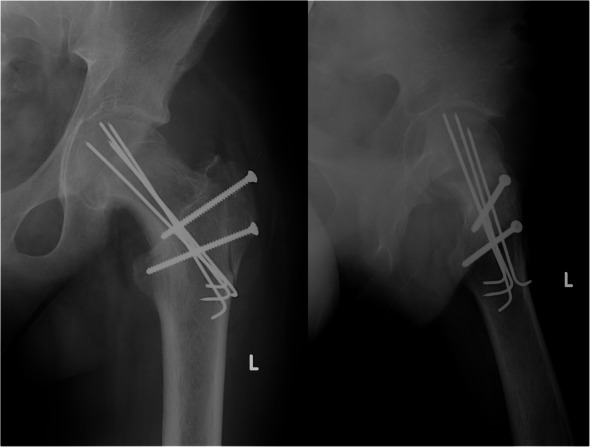
Postoperative hip XR anterior-posterior and axial views

Three months after the procedure, the patient presented to the emergency room with severe pain in the left hip joint. The ailment characterized as recurrent started about a year ago. A sudden increase in pain intensity occurred six months later and resulted in patient ambulation on crutches to prevent weight bearing on the affected limb. Physical examination revealed the Drehmann’s sign was positive, the left limb in the extension was rotated 45 degrees, all movements at the hip joint were excruciating. There were no vascular and nervous disturbances. The pelvis A-P and Lauenstein’s axial radiography and Computed Tomography of the pelvis were performed (Figs. 2, 3 and 4). Diagnostic imaging has showed bilateral opened epiphyseal cartilages of the femurs and SCFE on the left, affecting lateral and posterior part. The clinical and radiological assessment suggested proceeding with an open joint reconstruction with Dunn’s dislocation description by Ganz [9, 10] (Figs. 5, 6). The following day patient was placed in the upright position, and the rehabilitation started. After two more weeks patient was permitted to partially bear weight on the affected leg. Two months after the operation patient was able to fully bear weight on the affected limb. Three months after the operation patient regained full range of motion and walked without crutches (Table 1, Fig. 7).

**Table 1:** Range of motion and X-Ray parameters befora and 3 months after surgery

		Before the surgery	3 months after the surgery
Range of motion	flexion	50	130
	abduction	10	45
	adduction	0	15
	Internal rotation	−15	30
	External rotation	45	50
	Drehmann’s symptom	+	–
Radiological parameters	ET	75	0

ET – Southwick’s lateral epiphyseal-shaft angle

**Fig. 8:**
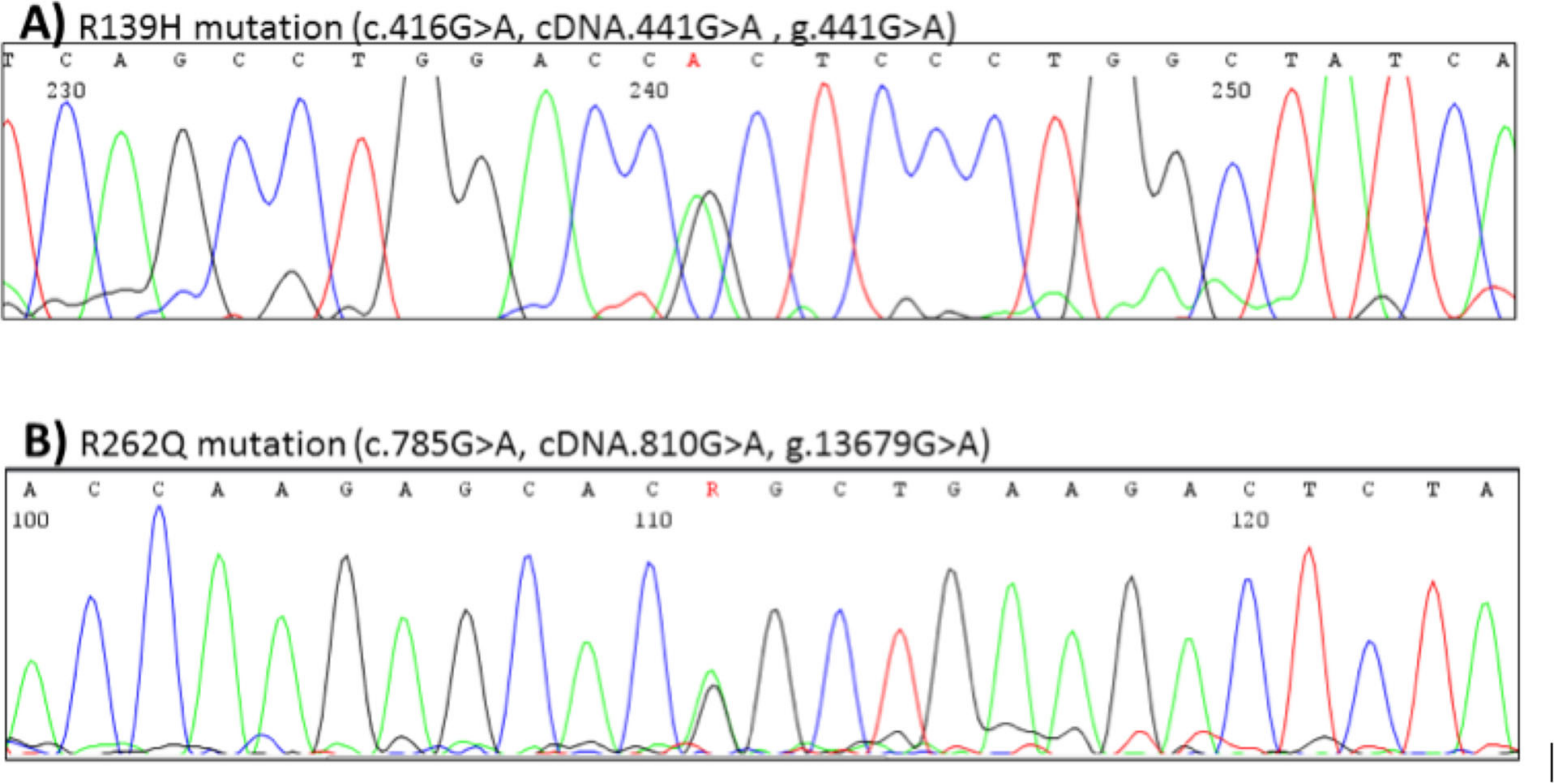
Direct sequencing of GNRHR gene and compound heterozygosity for R139H (**A**) and R262Q (**B**) mutations (depicted by an arrow) detected in patient’s DNA

**Fig. 9:**
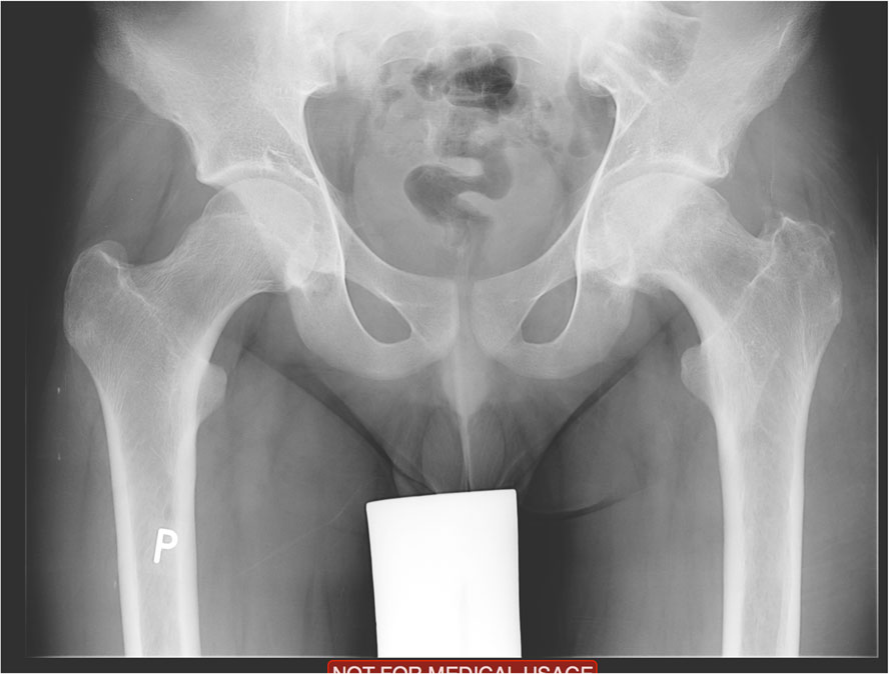
Postoperative XR axial plane

**Fig. 10:**
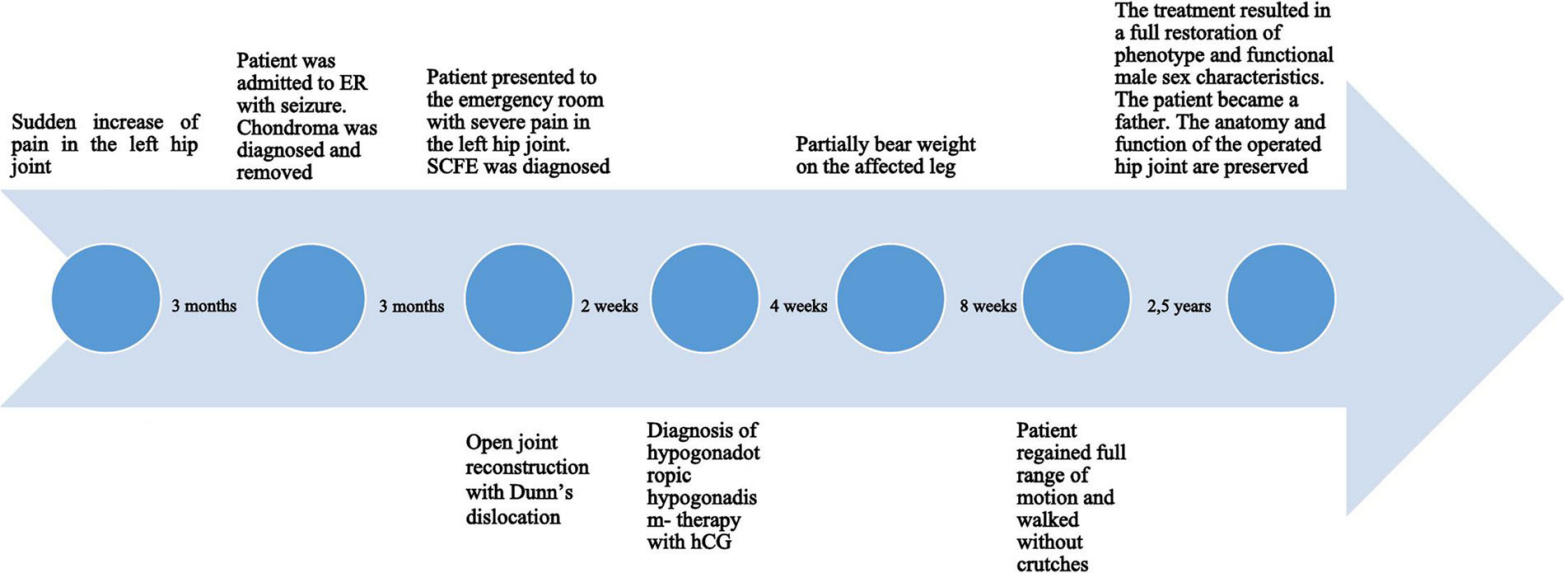
Patient treatment timeline

Endocrinological consultation was suggested due to clinical features of hypogonadism and revealed that the patient was 194 cm tall and his weight was 83 kg (BMI 22.05 kg/m2). His arm span was 197 cm. He presented features of delayed sexual maturation. Examination of external genitalia showed micropenis and testicular volume of 3.3 ml and 3.6 ml in the right and left testes, respectively (Tanner’s stage of 2). To our knowledge, these signs of delayed puberty were firstly observed during an endocrine consultation on SCFE. Laboratory tests revealed hypogonadotropic hypogonadism with no other features of pituitary insufficiency (Table 2). Normal pituitary gland was visualized in NMR. The bone age was 15 years. Dual-energy X-ray absorptiometry was performed, and osteoporosis was found (Z-score at lumbar spine − 3.4). Clinical findings were also reflected in a genetic test. Direct sequencing of gonadotropin-releasing hormone receptor (GNRHR, MIM:138850, ref.: NM_000406.2) revealed compound heterozygosity for two independent mutations R139H and R262Q (each allele is affected by one mutation, Fig. 8). The genetic status of a patient leads to the synthesis of altered GNRHR protein. The patient had a normal male karyotype (46XY), and other chromosomal rearrangements in the genome that could be responsible for the phenotype, were excluded. The treatment with human chorionic gonadotropin (hCG) was initiated two weeks following the surgery to achieve both masculinization and spermatogenesis. The treatment resulted in a complete restoration of phenotype and functional male sex characteristics. The patient became a father of a healthy daughter three years after treatment initiation. The anatomy and function of the operated hip joint are preserved (Fig. 9). Figure 10 presents patient treatment timeline.

**Table 2:** Serum hormones’ concentration

Hormone	Concentration	Norm range
FSH	0.7 mIU/ml	1.5–12.4
LH	0.3 mIU/ml	1.7–8.6
Testosterone	0.6 nmol/l	9.9–27.8
TSH	3.5 μIU/mL	0.27–4.20
FT4	18.57 pmol/l	11.5–21.0
FT3	5.58 pmol/l	3.93–7.70
PRL	251 μIU/ml	85–390
ACTH	57.62 pg/ml	7.2–63.3
GH	0.12 ng/ml	0.03–2.47
IGF-1	340 ng/ml	170–418

FSH - follicle-stimulating hormone, LH - luteinizing hormone, TSH - thyroid-stimulating hormone, FT3 - triiodothyronine (thyroid hormones), FT4 – thyroxine (thyroid hormones), PRL- prolactin, ACTH - adrenocorticotropic hormone, GH- growth hormone, IGF-1 - insulin-like growth factor 1

### Patients’ perspective

“*I had always felt different. My appearance and even the way I behaved had been very different from most my collegues. After I started the therapy, my body has changed, my mood, even my voice. After several months of the therapy I felt in love for the first time in my life, and I am a happy father of a wonderful girl.”*

## Discussion and conclusions

Although SCFE is a common disease of the hip joint it creates a big challenge to treat its subsequent deformation from the surgical perspective. Avascular necrosis (AVN) and chondrolysis occur more often in the group of patients operated on than in patients without surgical intervention or only with neck and head osteosynthesis in situ (without reduction of deformation) [11–13]. The osteosynthesis of the neck and head in situ causes deformation leading to earlier degenerative changes of the hip joint and results in a femoro-acetabular-impingement. Traditional procedures (e.g. Imhauser’s osteotomy, Southwick’s intertrochanteric osteotomy) are safe; however, correction of deformations only partially improves the joint function. Artrotomy became a safe procedure due to research made at Berne’s school for surgery dislocation of the hip joint [14, 15].

It is worth emphasizing that complete epiphyseal fusion occurs between 16 and 19 years old in European-American males [16]. Since our patient was 25 years old, the delay of bone age = was about 10 years.

Another fact that makes the described case unique is that SCFE mainly occurs in obese patients [3, 4], while our patientBMI was 22.05 kg / m2, which is the desired body weight.

Interesting is also the fact of late diagnosis (when full sexual maturity should have been achieved for several years) of delayed puberty. There is no doubt that this should have been diagnosed much earlier than at 25. We might speculate that the undiagnosed delayed puberty reflects the weakness of the health care system and cultural and social factors influencing an individual reaction to being different.

As was mentioned above, SCFE in adults is extremely rare and is always associated with endocrinopathy. To date, there are very few cases reported of SCFE in adults with panhypopituitarism, hypogonadotrophic hypogonadism, hypothyroidism, acromegaly [17–23]. Therefore, every patient diagnosed with SCFE in adulthood should undergo endocrinological assessment based on physical examination and laboratory tests. Our patient presented with a genetically caused autosomal recessive GNRH-related hypogonadotropic hypogonadism. It seems that the giant chondroma was an unrelated co-morbidity.

## Data Availability

Not applicable.

## Abbreviations

SCFE: Slipped capital femoral epiphysis
FAI: femoro-acetabular impingement
BMI: body mass index
MRI: Magnetic resonance imaging
DEXA: dual-energy x-ray absorptiometry
hCG: human chorionic gonadotropin
GNRHR: gonadotropin-releasing hormone receptor
FSH: follicle-stimulating hormone
LH: luteinizing hormone
TSH: thyroid-stimulating hormone
FT3: triiodothyronine (thyroid hormones)
FT4: thyroxine (thyroid hormones)
PRL: prolactin
ACTH: adrenocorticotropic hormone
GH: growth hormone
IGF-1: insulin-like growth factor 1
ET: Southwick’s lateral epiphyseal-shaft angle
